# *Tectus (Trochus) niloticus* search for suitable habitats can cause equivocal benefits of protection in village-based marine reserves

**DOI:** 10.1371/journal.pone.0176922

**Published:** 2017-05-02

**Authors:** Pascal Dumas, Jayven Ham, Rocky Kaku, Andrew William, Jeremie Kaltavara, Sompert Gereva, Marc Léopold

**Affiliations:** 1 IRD, UMR 9220 ENTROPIE, Nouméa, Nouvelle-Calédonie; 2 Laboratoire d’Excellence LABEX Corail, Perpignan, France; 3 Fisheries Department of Vanuatu, Port-Vila, Vanuatu; University of Sydney, AUSTRALIA

## Abstract

In the Pacific, the protection of coral reef resources is often achieved through the implementation of village-based marine reserves (VBMRs). While substantial fisheries benefits are often reported, results of quantitative approaches are controversial for benthic macroinvertebrates, whose life history traits may cause low congruence with protective measures implemented at non-ecologically relevant scales. This study investigated the structural and behavioral responses of the exploited topshell *Tectus niloticus* within a very small (0.2 km^2^) VBMR in Vanuatu, south Pacific. The results of underwater surveys and a nine-month tagging experiment emphasized contrasted, scale-dependent responses. At the reserve scale, our results failed to demonstrate any positive effect of protection after three years of closure. In contrast, abundance, density and biomass increased more than ten-fold in the southern part of the reserve, along with significantly larger (25%) individual sizes. The dispersal of tagged specimens was also consistently lower after 2, 4 and 9 months in the latter zone. Analyses of 17 substratum variables revealed a marked small-scale patchiness delineating contrasted benthic microhabitats, the distribution of which closely matched that of trochus. We advocate that i) VBMRs have inherently unequal ecological potentials for protecting and managing highly habitat-dependent species such as trochus; ii) ‘success’ or ‘failure’ is to a certain extent pre-determined by the trajectory of species-specific microhabitats, which may outreach protection effects. This has strong implications in the Pacific where the location and size of reserves primarily depends upon marine tenure, and communities have little flexibility in setting reserve boundaries.

## Introduction

The topshell *Tectus niloticus* or “trochus” is a large, heavily-targeted coral reef gastropod found in the tropical and subtropical waters of the Pacific and Indian Oceans, whose high value and non-perishable quality make it an attractive source of income for isolated island communities [1–3]. Following a rapid geographical extension across the Pacific from multiple transplantations in the years 1930–1940, the rise in global demand of trochus shells on the world market quickly raised serious concerns about the preservation of this traditional resource [4–7]. The historical evidence of trochus vulnerability to over-harvesting, the use of increasingly restrictive fisheries regulations and improved aquaculture-derived reseeding actions have not prevented severe stock collapse in most Pacific countries, sometimes to the point of local extirpation [8–10].

The creation of no-take marine reserves or “sanctuaries” allowing significant increase in broodstock density is one of the oldest claims made by reef ecologists to support the self-replenishment of depleted populations [11, 12]. For trochus, rapid growth, early sexual maturity and limited larval dispersal constitute favorable life-history traits that are expected to enhance reproductive success in reserves and help to counterbalance Allee effects, at least at certain scales [12, 13]. In many Pacific countries, the protection of trochus or other valuable macroinvertebrates such as giant clams, green snails or sea cucumbers is often achieved through the implementation of village-based marine reserves (VBMRs). Such reserves are rooted in traditional marine resource management; since the 1990s they have received growing support at both national and international levels to address conservation objectives and short-term, local socioeconomic needs [14, 15]. They are implemented and managed by the local communities, generally with the advisory support of national government agencies and/or non-governmental organizations [16]. Recent studies indicate that VBMRs are generally perceived as effective by stakeholders, which was supported in certain cases by observations of significant increase in the abundance and/or size of target species inside the reserves, mostly fish [17–19]. Paradoxically, despite a large amount of grey literature, few quantitative studies have specifically addressed their efficiency to significantly restore and/or enhance major macroinvertebrate resources such as trochus [20]. Whether such tools might allow trochus populations to remain protected long enough before significant ecological benefits become visible on a larger scale and timeframe is still unclear.

In most VBMRs, protection effects may be challenged by periodic harvest, whose unpredictable occurrence and intensity reflects opportunistic community responses to a variety of social, cultural or economic drivers, rather than long-term conservation concerns [21]. Spatial scale is another issue that challenges the effectiveness of marine reserves in the Pacific [22]. VBMRs are usually limited in size with respect to marine tenure, which may not allow a significant proportion of trochus population to remain protected from harvest if their displacement range exceeds the no-take zone [23]. Moreover, recent studies highlighted that the efficiency of reserves can be modulated by small-scale heterogeneity in the reef habitat, in particular for sedentary macroinvertebrates whose distribution is better captured at small, e.g. metric scales [24, 25]. Benefits of protection may be obscured by behavioural responses to the unequal availability of suitable habitats, in particular the displacements of invertebrates within and across the reserve boundaries. Yet, despite physiological evidence of limited locomotory ability, very little is known on the displacement behaviour of this species [26].

In this study, we investigated the synergistic effects of habitat and protection on the population structure and the behavioural responses of the topshell *Tectus niloticus*. Along with small-scale habitat structure, the abundance, density and biomass of trochus populations were surveyed inside and outside a very small (<0.5 km^2^ reef) village-based marine reserve located in Vanuatu, South Pacific. The habitat was characterized at small scale using 17 local habitat variables (sediment characteristics, reef structuring species, coral growth forms) derived from an underwater photographic approach. Trochus abundance, density and size structure were measured underwater with the support of local villagers. The displacement behaviour of adult trochus was investigated using 100 tagged individuals that were released at four locations inside the reserve and periodically assessed over a 9-months period.

## Materials and methods

### Study area

**Fig 1:**
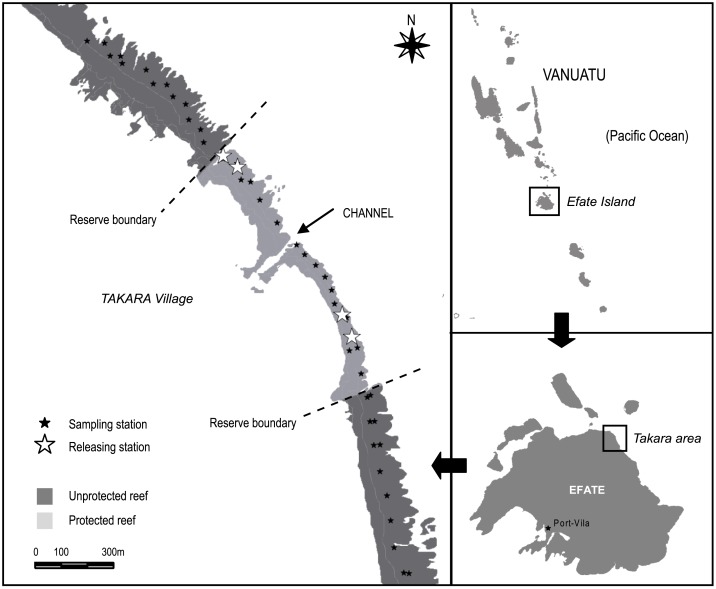
Location of the study area in North Efate, Vanuatu (South Pacific). Boundaries of the Takara marine reserve, reef sampling and trochus tagging release stations are indicated.

The study was undertaken on the northern coast of Efate island, along a 2.3 km linear, shallow fringing reef located in front of the Takara village (17°32.407 S, 168°27.231E) (Fig 1). Fishing activities were controlled by the villagers through the implementation of a locally-managed marine reserve or ‘tabu area’ (Takara VBMR hereafter). The shoreline-bounded VBMR was established in 2008. It covers a 1.1 km^2^ area in front of the village, including a fringing reef area of 0.2 km^2^ with a linear coastal extension of 0.9 km. In the central zone of the reserve, a narrow channel (3-8m deep; 15m wide) splits the reef area into two zones. The reserve was managed by a total fishing ban on all fish and invertebrate species, which could be eventually lifted to allow limited harvest by the community for specific events.

### Trochus census

The study focused on the trochus shell (*Tectus niloticus*), that has been harvested throughout Vanuatu for commercial purpose since the 1800s. Following generalized stock depletion, the minimum harvestable size has been set at 9 cm (basal diameter) by a 1982 fisheries act. The sampling surveys occurred in December 2011 after permission was obtained from the customary land owners. The stations were preselected using geomorphological classification and available habitat maps [27], so as to exhibit habitat characteristics suitable for trochus. We retained 41 ocean-exposed, fringing reef stations (16 inside vs. 25 outside the reserve) composed of an association of rocky substratum and corals in zones subject to moderate to high wave action. Distance between the stations of approximately 50m; depth range was 1–4m. On each station, a randomly selected 50m transect was materialized by a color-marked survey tape attached to the substratum. All transects were geolocalized by marking both extremities with a handheld Garmin GPSMap60Cx in an underwater housing. Data was collected by a team of two snorkellers swimming simultaneously along both sides of the transect line, each surveying a 2m-wide corridor. All trochus detected along the corridors were counted and individually measured to the nearest mm (shell diameter) using calipers. Following Bour [4], individual biomass was estimated using the following relationship:

Ln (Ws) = −8.62 + 3.09 Ln (D)

Where:

W_s_: shell biomass (in g)

D: shell diameter (in cm)

### Habitat description

A benthic substratum survey was performed simultaneously to investigate the effects of fine-scale habitat on trochus distribution and movements. At each of the 41 stations, sediment type and substratum coverage variables were estimated using a standardized photographic method. Pictures were taken from the surface using a standard digital 8 Mpixels CanonS90 camera in underwater housing, oriented perpendicular to the substratum. A total of 25 pictures per transect (one shot every two meters) were recorded and subsequently imported into a dedicated image analysis program (CPCe software, [28]). Seventeen local habitat variables were considered, related to sediment type and substratum coverage by sessile macroorganisms (Table 1). Surface estimates expressed in percentage covers were derived from random stratified point count techniques using a 9 points.m^-2^ ratio, to ensure reliable habitat profiles [29]. Percentage covers were then aggregated at the transect level.

**Table 1:** Habitat variables referring to sediment type, substratum coverage used for habitat characterization in the study area in North Efate, Vanuatu (South Pacific).

Sediment type	Substratum coverage
Mud	Branching corals
Sand	Digitate corals
Rubble	Tabular corals
Boulders (<100 cm)	Massive corals
Dead corals	Submassive corals
Bedrock	Foliose corals
	Encrusting corals
	Soft corals (Alcyonarians)
	Fire corals (Milleporidae)
	Seagrass
	Macroalgae

### Trochus tagging

A tagging survey was initiated in December 2011 in the study area. A total of 100 adult specimens (size range 82–145 mm) were collected inside the reserve by five experienced snorkelers. Individual, numbered soft plastic tags of 2x1cm were screwed to the outer margin of the shell after a 3 mm hole was carefully drilled through each shell lip with a masonry drill bit. Each tag was secured by an aluminum washer. The tagged specimens were released on the same day in four random locations inside the reserve: two in the southern zone (R1, R2) and two in the northern zone (R3, R4) (Fig 1). Snorkelers carefully replaced the tagged trochus under natural shelters (crevices, tabular corals etc.) to avoid immediate post-release predation. The position of the tagged specimens was further recorded after 2, 4 and 9 months to investigate trochus displacement. On these occasions, a team of 5 snorkelers carefully searched the area during approximately four hours, swimming in concentric circles around the release locations to locate the tagged trochus. In order to avoid displacing and stressing the animals, the specimens sighted had their position signaled by a surface buoy, connected to a 1kg weight deposited on the substratum right next to the animal. The position of each buoy was recorded by a snorkeler holding a handheld Garmin GPSMap60Cx placed in an underwater housing, pulling the string so that the buoy was vertical to the tagged specimen.

### Data analysis

The influence of protection on trochus populations was first assessed globally. Density, biomass and size data were compared between reserve and open stations using non-parametric Mann—Whitney U-tests, as data could not be satisfactorily normalized. Small-scale (within-reserve) spatial patterns and habitat-related effects were further investigated after data were integrated into a geographical information system and plotted. A multidimensional similarity matrix based upon the calculation of Euclidian distances between all the stations was built using the substratum variables. Transects were then ordinated using Principal Coordinates analysis (PCO) in order to establish a multifactorial typology of the habitat; groups (clusters) of stations sharing similar habitat features were constituted using the similarity coefficients. Between-group discrimination was tested using PERMANOVA analyses performed on the habitat variables, with subsequent pair-wise tests by permutation using 9999 permutations. Trochus density and biomass were then plotted on the resulting diagram in order to visually investigate species-habitat relationships. Differences in abundance, density, biomass and size of trochus were tested using non-parametric Kruskal—Wallis tests.

For the tagging survey, the recapture rate was calculated at each release location and each survey as follows:

$$Rt\left(\%\right)=\frac{Nt}{Ni}\times 100$$

where:

R_t_: recapture rate in percent at time t

N_t_: number of alive tagged specimens recaptured at time t

N_i_: number of tagged specimens initially released

For each recaptured individual, the displacement was expressed as the linear distance in meters between its recapture position and the original release location. The behavioural responses to reef habitat within the reserve (northern vs. southern zone) and across time (after 2, 4, 9 months) were tested using displacement distances with a two-way ANOVA (zone x date, fixed factors), after data were successfully log-transformed to meet the assumption of normality. All analyses were performed using Statistica v.6 and Primer v.6 with PERMANOVA add-on packages.

## Results

### Trochus population patterns

In the Takara area, trochus were generally found in low to medium abundances, ranging from 0 to 9 individuals per transect, i.e. 0 to 450 indiv.ha^-1^ (mean 73.2 indiv.ha^-1^). Size data encompassed a significant range of size classes from juveniles to adults, with shell diameters between 40 and 143 mm. Mean biomass was 0.54 kg per transect (26.9 kg.ha^-1^), with 60% specimens of legal harvestable size (mean harvestable biomass 0.30 kg.transect^-1^, i.e. 15.2 kg.ha^-1^).

When habitat was not considered, the effects of protection could not be clearly discerned between the reserve and the adjacent unprotected reef areas for most population variables (Table 2). Density and biomass were greater inside the reserve (mean density 90.6 indiv.ha^-1^ and 62.0 indiv.ha^-1^ inside vs outside the reserve), but the results were not statistically significant (Mann—Whitney U-test, N.S. in both cases). Similar patterns were found for the legal-sized fraction of the population (Mann—Whitney U-test, N.S. for both harvestable density and harvestable biomass). In contrast, slightly larger trochus were observed inside the reserve (mean diameter 113.5 mm and 95.2 mm inside vs. outside the reserve; Mann—Whitney U-test, p<0.05).

**Table 2:** Effects of protection on trochus (*Tectus niloticus*) populations in the Takara reserve, North Efate, Vanuatu. Means (±SE) for abundance, biomass and individual size inside vs. outside the marine reserve. Results of Mann Whitney’s U test: * p<0.05.

	Inside reserve	Outside reserve	P
Nb transects	16	25	-
Total abundance (nb.transect^-1^)	1.8 (0.7)	1.2 (0.3)	0.91 (N.S.)
Harvestable abundance (nb.transect^-1^)	1.4 (0.6)	0.5 (0.2)	0.19 (N.S.)
Total biomass (kg.transect^-1^)	0.8 (0.3)	0.4 (0.1)	0.60 (N.S.)
Harvestable biomass (kg. transect^-1^)	0.5 (0.2)	0.1 (0.04)	0.21 (N.S.)
Individual size (mm)	113.4 (3.5)	95.2 (5.3)	0.03 (*)

### Habitat patterns

**Fig 2:**
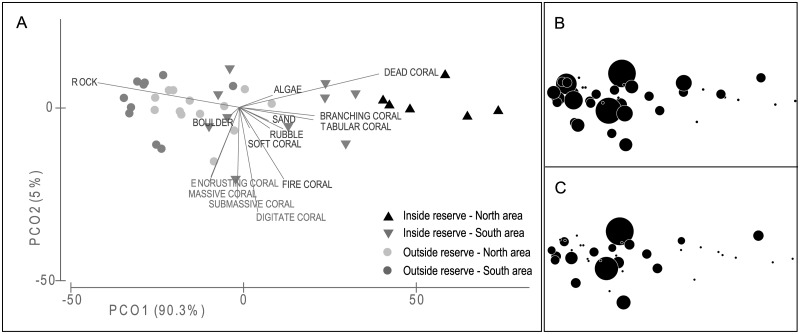
Habitat typology in the study area. Results of Principal Coordinates Analysis (PCO) of the reef stations based upon benthic habitat variables: (a) station plot by zone (North, South) vs. protection status (inside, outside the reserve). Same plots with black circles proportional to: (b) the density and (c) the legal-sized biomass of trochus topshell (*T*. *niloticus*).

In the area, the benthic habitat was patchy and constituted of rocky substratum (mean cover 85.5±8.9%) with moderate living coral cover (13.5±9.0%) and very low macrophytes, algae or fine sediment (<1%). Ordination of the substratum variables by PCO highlighted a major gradient across the study area, delineating contrasted small-scale habitats (‘microhabitats’ hereafter) inside and outside the reserve (PERMANOVA with subsequent between-group pair-wise tests, Pseudo-F = 36.96, p<0.001 for all groups). Within the reserve, the PCO plot mainly discriminated between stations located on either side (north and south) of the channel, based upon their topographical complexity (Fig 2). The microhabitat in the northern zone was characterized by significant cover of non-eroded, dead corals (mean 60.1±8.1%, correlations with axis PCO1: 0.97) associated with patches of living corals (mean 17.1±6.2%), particularly tridimensional growth forms such as branching, tabular or fire corals (correlations with PCO1 from 0.31 to 0.52). Flat, rocky surfaces were not dominant (mean 19.7±11.1%). In contrast, stations from the southern zone of the reserve exhibited eroded, bedrock-dominated microhabitats (mean 52.1±12.8%, correlation with PCO1: -0.99) with limited coral cover, either living (mean 13.7±11.9%) or dead (mean 28.4±13.7%), and a subsequent reduced structural complexity. This trend was further noticeable in the adjacent reef areas outside the reserve where habitat was nearly almost exclusively composed of flat, bare rock surfaces (mean 74.7±10.2%), especially in the southern open area.

### Reserve effects with respect to small-scale habitat

**Fig 3:**
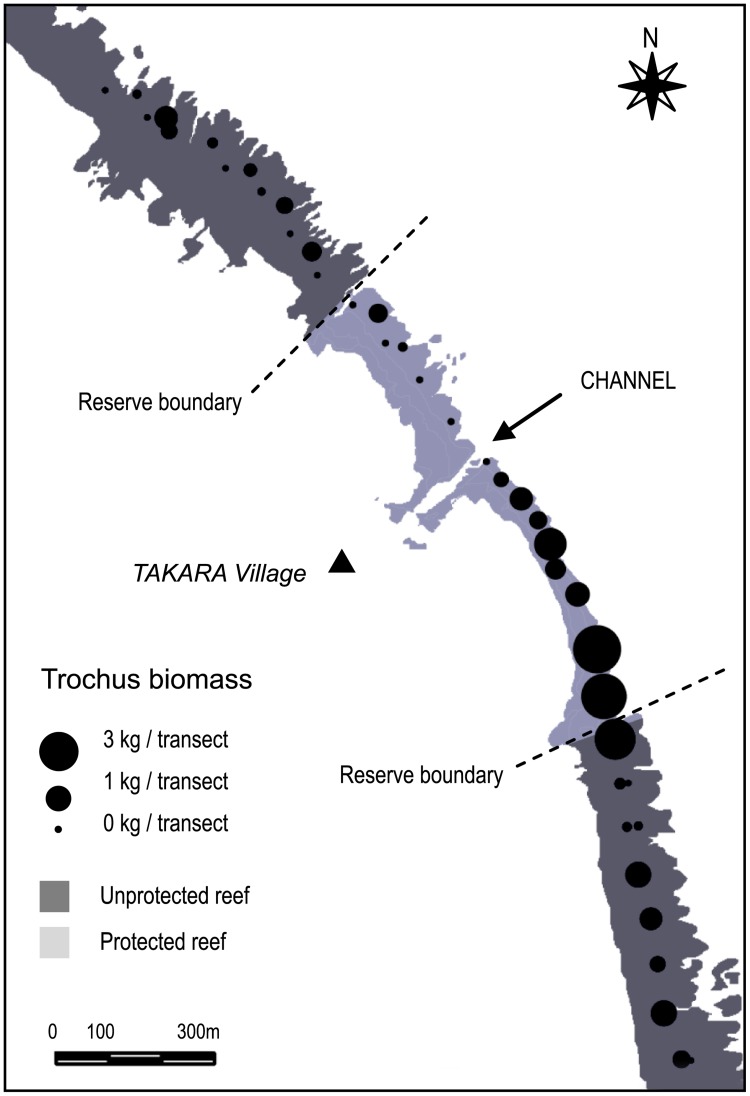
Spatial distribution of trochus biomass in the study area. Mean biomass (kg per station) within and outside the Takara reserve.

**Fig 4:**
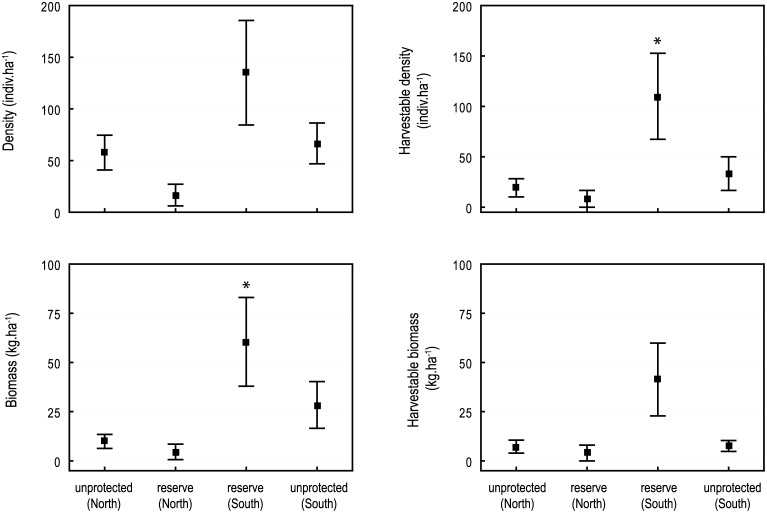
Comparison of trochus populations within and outside the Takara reserve. Total and legal-sized fraction of density and biomass (mean±SE). Results of Kruskal-Wallis tests: * p<0.05.

Results emphasized spatially-contrasted trochus distribution patterns that closely matched those of reef microhabitats (Fig 3). The total biomass was thirteen-fold larger in the southern zone compared to the northern zone of the reserve, where trochus were nearly absent (mean 1.21 vs. 0.09 kg.transect^-1^; Kruskal—Wallis test, n = 41, p<0.05). Very similar patterns were observed for the harvestable fraction of the population, with a ten-fold increase in harvestable density inside the southern part of the reserve (110 indiv.ha^-1^ vs. 8.33 indiv.ha^-1^, Kruskal—Wallis, p<0.05). On the other hand, abundance, density and biomass estimates were generally low in the northern side of the reserve and in the open stations, despite marked trends towards higher values in the unprotected reefs adjacent to the reserve (Kruskal—Wallis, N.S. in all cases) (Fig 4).

### Trochus dispersal

**Fig 5:**
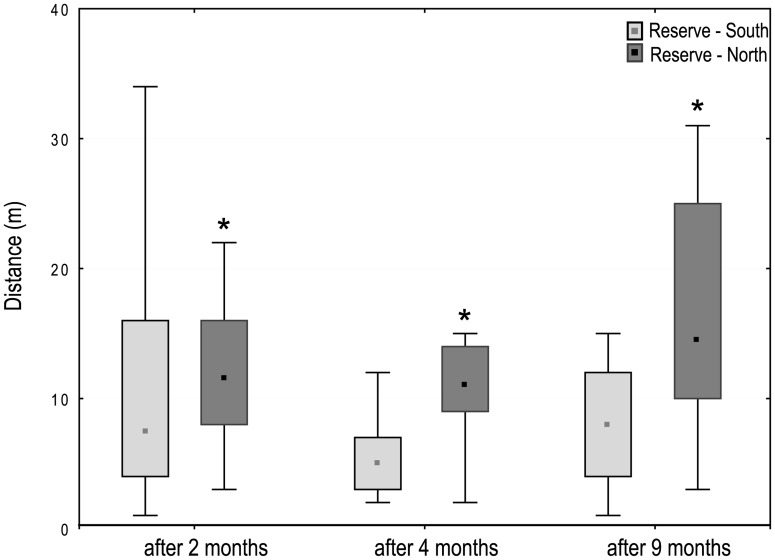
Mean linear distances travelled by tagged trochus within the Takara reserve. Median with 25–75% percentile and results of two-way ANOVA after 2, 4 and 9 months (* p< 0.05).

Tagged trochus generally exhibited high recapture rates across recapture surveys, whatever the release location. Recapture ranged from 51% to 93% and clearly decreased with time, with an average of 80%, 62% and 53% of tagged individuals recaptured in the reserve after 2, 4 and 9 months, respectively. The individual distances measured ranged from 1 to 49m; on average individuals were recaptured 6.9 ± 6.8m (R1), 10.3± 7.9m (R2), 12.9 ± 7.8m (R3) and 15.7 ± 9.6m (R4) from their respective release locations across the recapture surveys. Marked patterns were observed within the reserve: displacements were significantly greater in the northern part when compared to the southern part of the reserve (mean 13.7m vs. 9.1m respectively; 2-way ANOVA, zone: F = 32.178, p<0.001). This difference was consistent over time (2-way ANOVA, date: F = 2.18, N.S. and interaction zone*date: F = 0.927, N.S.) (Fig 5).

## Discussion

### VBMR, an effective tool to manage trochus resources?

Despite a recent upsurge in the creation of village-based marine reserves in the South Pacific, only limited information is available on their capacity to maintain and/or restore resources deemed vulnerable to exploitation [30]. This is particularly true for benthic macroinvertebrates which constitute major subsistence and/or economic species across the Indo-Pacific [2, 31]. While communities frequently report rapid and substantial fisheries benefits within or closely around reserves boundaries, results of quantitative approaches are less clear [14]. In this case study, we investigated the quantitative effects of VMBR on the highly prized topshell trochus (*Tectus niloticus)*. Trochus constitute spatially explicit structured resources whose heterogeneous distribution changes within small distances, under the combined—and sometimes antagonistic—influence of environmental conditions, biological interactions (intra/interspecific) and anthropogenic activities [32–34]. At the full reserve scale, which is usually the only considered in conservation or fishery management approaches, our results failed to demonstrate any positive effect of protection. At the time of the study (after 3 years of closure), the mean abundance, density and biomass of *T*. *niloticus* were generally low and did not overall differ between protected and unprotected areas. The only significant effect was observed on the size distributions, with slightly larger (19%), i.e. older individuals found inside the reserve. Cases studies where protection had no measurable impacts on benthic macroinvertebrates were similarly reported in Vanuatu [35] or in the Solomon islands [3], the failure of which being mainly attributed to interactions between anthropogenic factors (including closure regimes, local governance and various socioeconomic features) and the biological responses of the target species. On the other hand, positive effects were documented in the neighboring Emao island where a three-fold increase in trochus density was measured in a similar, yet even smaller reserve after four years of protection [20]. However, the authors acknowledged that the observed difference may result from short-term population enhancement (e.g. due to the active translocation of adult trochus into the reserve by the villagers), rather than from natural recovery processes (including effective reproduction, recruitment, growth) that would sustain trochus resources in the longer term.

Along with identifying the processes responsible for shaping the trochus distributions, our results also raised the issue of sampling effort required to detect and quantify subtle or highly spatially-constrained effects. There is little need to re-iterate that the lack of robust sampling design and sufficient statistical power (e.g. due to high natural variability and multi-scale interactions of the studied factors, lack of spatial and/or temporal replication and more generally to limited sample sizes) makes it difficult to validate the information provided by ecological indicators (e.g. [36–40]). This is often observed in VBMRs, where UVC-based surveys are generally conducted with low spatial replication due to spatial, financial and logistical constraints [41]. Here, the sampling effort was enough to document the prevailing trends in trochus populations across the study area, but was clearly insufficient to quantify the effect sizes in low population density areas which exhibited a high variance for all the trochus indicators. Reducing this uncertainty is challenging, yet crucial to provide communities with operational recommendations in terms of management. Increasing sampling power could be in particular achieved by i) increasing the sampling effort (at least to a certain extent, as too many stations may entail a risk of spatial autocorrelation in the smallest VBMRs); and ii) using spatial stratification, where sample allocation would explicitly encompass the distribution of the available microhabitats in the study area.

### Equivocal effects of VBMR: the importance of ecological scale

While it is widely recognized that effects of VBMR are highly context-specific [19], very few scientific papers have specifically addressed the underlying ecological mechanisms that condition and eventually undermine their efficiency [42]. This is especially true for highly sedentary or sessile macroinvertebrates, whose specific life history traits (marked substratum relation, reduced adult mobility, high larval spillover) may cause low congruence with protective measures implemented at non-ecologically relevant scales. For example, equivocal effects of protection were recently documented for giant clams and trochus in a network of New Caledonian marine protected areas, as a result of high levels of environmental variability at the microhabitat scale [43]. In our study, highly significant effects of protection were detected in the southern zone of the reserve where trochus population density and biomass increased more than ten-fold, along with a marked increase in individual sizes (+25%). On the other hand, trochus were virtually absent in the northern part of the reserve, despite apparently similar hydrodynamics and large-scale geomorphological reef characteristics. This discrepancy could theoretically result from human activities being unequally distributed: along with periodic openings of the closure, the translocation of adult trochus from surrounding reefs by the fishers is a common practice in the area. However, informal interviews with local fishers and community leaders did not provide evidence of any differential management practices (occurrence of openings, harvest methods, catch limitations, enforcement capacity, etc.) or particular events (translocation, illegal fishing) affecting a specific part of the reserve.

In contrast, our results revealed the presence of a marked, intra-reef habitat heterogeneity linked to the relative distribution of benthic structuring elements: bare substratum, dead corals and a variety of coral growth forms across the area. A marked spatial heterogeneity is a distinctive characteristic of coral reefs ecosystems, where it can be realized from small, intra-reef (typically 10^0^–10^2^m or lower) to large, geomorphologic scales (whole reefs, 10^2^m or above) [44–46]. In this study, we showed that small-scale substratum patchiness delineated contrasted benthic microhabitats, the distribution of which matched that of trochus populations. In particular, the dense populations harbored in the southern part of the VBMR could be associated with low topographically complex reef areas composed of eroded, almost bare rock pavement balanced with very little corals, macroalgae, macrophytes or other reef-covering species. These results are consistent with the classification recently developed for trochus in the fringing reefs of New Caledonia, where the highest population densities were significantly associated with three of the six benthic microhabitat categories described [43]. Despite harvest pressure, these preferences were further noticeable in the adjacent, bedrock-dominated unprotected reefs where trochus density and biomass eventually reached higher values than inside the northern part of the reserve. Unfortunately, the combination of low sampling replication, patchy trochus distribution and heterogeneous harvest pressure entails inadequate statistical power to discriminate between environmental and anthropogenic influences in these areas. While they will require further testing using more robust sampling procedures, these findings confirm that habitat-related effects may in certain cases outreach fishing effects on trochus populations, and that open areas may even have higher potential for population restoration than reserves.

### Behavioral responses of trochus to microhabitat structure

The recognition of strong species-habitat relationships for trochus is not new [47]: adult trochus were known to live preferentially in areas with extensive cover of rubble, rock/algal pavement and/or corals in the windward intertidal and shallow subtidal zones of coral reefs [4, 5, 48]. While inferring accurate activity from non-continuous monitoring is hazardous, our results further suggest that reef structural complexity represents a major driver of habitat use by trochus. Travel distances were significantly shorter in the southern, bedrock-dominated part of the reserve; conversely the dispersion was higher in the more structurally-complex, northern area. This was consistently observed across the 9-month tagging period and may indicate contrasted behavioral responses linked to trochus ecology, in particular locomotion and nutrition [26]. It has been demonstrated that locomotion is influenced by species-specific life-history traits and the topographic complexity of habitats [49, 50]. *T*. *niloticus* is a large benthic species reaching up to 15cm in diameter. It crawls onto the substratum using its large, muscular foot and may therefore avoid areas with complex tridimensional features (dense assemblages of branching, tabular, fire corals etc.) where it could be impeded by its shell. Contrasted behaviour in relation to benthic topography was described for the benthic gastropods *Austrocochlea porcata*, *Nerita atramentosa*, and *Bemicium nanum*, with smaller travel distances being recorded over complex rocky substrata [51]. For trochus, the longer distances consistently recorded in the northern area may suggest a greater locomotor activity displayed by individuals released in non-optimal microhabitats, actively searching for more suitable conditions. Microhabitat selection may be further reinforced by spatial variations in other environmental and/or biological factors that we did not measure during this study, in particular food availability. *T*. *niloticus* is a grazing herbivore and detritivore that uses its rasp-like radula to feed on algal biomass, in particular on green turf algae that preferentially grow in rocky areas with unobstructed exposure to surf [5]. The longer travel distances recorded in the northern, coral-dominated zone may indicate an increased foraging behavior, as it has been showed that decrease in micro-algal food can increase dispersal in marine gastropods [52, 53]. While these dispersion patterns were consistently observed over a significant (9 months) period, some inherent limitations of the study should be kept in mind. The generality of these findings may in particular be limited by our current (technical) inability to continuously record trochus trajectories on the long term, and/or by the lack of temporal replication of this study. Marked ontogenic changes are also likely to influence locomotion, as it was recently demonstrated that younger individuals displayed greater activity reflected by more intense and longer movements during the night [26].

### Encompassing small-scale habitat in conservation and management decisions

There is a rising trend to put emphasis on the social processes that drive conservation, in particular in Pacific island countries where community-based initiatives are increasingly addressed and analyzed in the light of knowledge, governance, compliance or management strategies [54, 55]. This study argues that, beyond anthropic aspects, VBMRs have inherently unequal *ecological* potentials for restoring and/or maintaining the benthic resources. Consequently, benefits -and more generally ‘success’ or ‘failure’- are to a certain extent pre-determined by the availability and trajectory of species-specific microhabitats, whose distributions are highly variable spatially. Conversely, it reminds us ecologists that the greatest care is needed to ensure that protection effects are not confounded by other factors, due to inadequate sampling design [56–59]. Ideally, this would include appropriate replication of sites with comparable microhabitat features inside and outside the VBRMs—which, in practice, will most likely require conducting specific habitat surveys prior to any other step, along with better stratification and/or more intensive sampling effort. It must also be kept in mind that microhabitat distribution may also vary temporally, as a result of dynamic redistribution in the coral species, growth forms, cover and subsequent changes in topographic complexity linked to a variety of environmental or anthropogenic factors [47, 48]. Significant alterations in the composition of coral assemblages were for instance observed in the Takara VBMR, as a consequence of storm surges and strong wave action when the tropical cyclone PAM hit the island in March 2015 [60].

These findings have strong implications in Vanuatu and in the neighbor countries, where the location and size of reserves primarily depends upon the enforcement of traditional access rights, and where communities may have very limited options to eventually relocate ‘unsuccessful’ reserves. Compared to westernized marine protected areas, VBMRs are typically smaller in size by one or two orders of magnitude (e.g. 0.1 – 1km^2^, [61]) and established in the vicinity of populated areas. Most communities therefore have little flexibility in setting reserve boundaries, which may undermines their capacity to efficiently protect highly habitat-dependent species such as trochus or other sedentary, low mobility benthic species. Our results further show that the apparent effects of protection are strongly dependent on the spatial scale at which they are investigated, even within very small, seemingly homogeneous geomorphologic units. Explicitly incorporating this dimension may help reduce the potential gap between the social expectations and the observed biological outcomes of VBMRs, a valuable step to better inform community-based management strategies. But communicating these concepts to the local communities is far from evident, in particular when social, economic or cultural constraints leave them with few alternatives [62].

The relative importance of protecting species versus habitat continues to attract some debate among scientists [63]. Despite continuous progress, relevantly scaling and mapping biological resources is still challenging, particularly in coral reefs which require specific sampling and analytic approaches across multiple scales [64]. Along with more traditional *in situ* approaches, the distribution of economically-important macroinvertebrates is increasingly investigated using aerial and satellite imagery, as a result of a rising demand for integrative tools operating at larger spatial scales (e.g. [65, 66]). Keeping in mind the overarching role of small-scale habitat factors in shaping species distribution patterns, combining these approaches looks a promising way towards more operational conservation of valuable benthic macroinvertebrate resources in the Pacific.

## References

[1] Pakoa K, Friedman K, Damlamian H, Tardy E. The status of the trochus (*Trochus niloticus*) resource in Tongatapu lagoon and recommendations for management. Noumea: SPC, 2009.

[2] Dalzell P, Adams TJH, Polunin NVC. Coastal Fisheries in the Pacific Islands. Oceanography and Marine Biology. 1996;34:395–531.

[3] Foale S. Assessment and management of the Trochus Fishery at West Nggela, Solomon Islands: an interdisciplinary approach. Ocean & Coastal Management. 1998;40(2–3):187–205.

[4] Bour W. Biologie, écologie, exploitation et gestion rationnelle des trocas (*Trochus niloticus* L.) de Nouvelle Calédonie. Nouméa: ORSTOM; 1989.

[5] Nash WJ. Trochus In: Wright A, Hill L, editors. Nearshore marine resources of the South Pacific; 1993 pp. 451–96.

[6] Heslinga GA, Hillmann A. Hatchery Culture of the Commercial Top Snail *Trochus Niloticus* in Palau, Caroline Islands. Aquaculture. 1981; 22(1–2):35–43.

[7] Gillett RD. Fisheries in the economies of the Pacific Island countries and territories. Mandaluyong City, Metro Manila, Philippines: Asian Development Bank; 2009 483 pp.

[8] Purcell SW. Management options for restocked trochus fisheries In: Leber KM, Kitada S, Blakenship HL, Svasand T, editors. Stock Enhancement and Sea Ranching: Developments, Pitfalls and Opportunities. Oxford: Balckwell; 2004 pp. 233–43.

[9] Amos M. Combination of fisheries management regulation, traditionally based management and wild stock enhancement using hatchery reared trochus juveniles as a precautionary management principle for *Trochus niloticus* resources in Vanuatu. Noumea, New Caledonia: 1995.

[10] Purcell SW, Amos MJ, Pakoa K. Releases of cultured sub-adult *Trochus niloticus* generate broodstock for fishery replenishment in Vanuatu. Fish Res. 2004;67(3):329–33.

[11] McGowan J. The Trochus fishery of the Trust Territory of the Pacific Islands. Saipan: 1958.

[12] Heslinga GA, Orak O, Ngiramengior M. Coral-Reef Sanctuaries for Trochus Shells. Mar Fish Rev. 1984;46(4):73–80.

[13] Foale S. Appraising the resilience of trochus and other nearshore artisanal fisheries in the Western Pacific. SPC Trochus Information Bulletin. 2008;14:12–6.

[14] Govan H. Achieving the potential of locally managed marine areas in the South Pacific. SPC Traditional Marine Resource Management and Knowledge Information Bulletin. 2009;25:16–25.

[15] Johannes RE. The renaissance of community-based marine resource management in Oceania. Annu Rev Ecol Syst. 2002;33:317–40.

[16] Leopold M, Beckensteiner J, Kaltavara J, Raubani J, Caillon S. Community-based management of near-shore fisheries in Vanuatu: What works? Mar Policy. 2013;42:167–76.

[17] Cinner JE, Marnane MJ, McClanahan TR. Conservation and community benefits from traditional coral reef management at Ahus Island, Papua New Guinea. Conserv Biol. 2005;19(6):1714–23.

[18] McClanahan TR, Marnane MJ, Cinner JE, Kiene WE. A comparison of marine protected areas and alternative approaches to coral-reef management. Curr Biol. 2006;16(14):1408–13. 10.1016/j.cub.2006.05.062 16860739

[19] Jupiter SD, Egli DP. Ecosystem-Based Management in Fiji: Successes and Challenges after Five Years of Implementation. Journal of Marine Biology. 2011;2011:1–14.

[20] Dumas P, Jimenez H, Leopold M, Petro G, Jimmy R. Effectiveness of village-based marine reserves on reef invertebrates in Emau, Vanuatu. Environ Conserv. 2010;37(3):364–72.

[21] Cohen PJ, Foale SJ. Sustaining small-scale fisheries with periodically harvested marine reserves. Mar Policy. 2013;37:278–87.

[22] Foale S, Manele B. Social and political barriers to the use of Marine Protected Areas for conservation and fishery management in Melanesia. Asia Pacific Viewpoint. 2004;45(3):373–86.

[23] Chapman MR, Kramer DL. Movements of Fishes Within and Among Fringing Coral Reefs in Barbados. Environ Biol Fish. 2000;57(1):11–24.

[24] English S, Wilkinson CR, Baker V. Survey manual for tropical marine resources. Townsville: Australian Institute of Marine Science; 1997.

[25] Dumas P, Kulbicki M, Chifflet S, Fichez R, Ferraris J. Environmental factors influencing urchin spatial distributions on disturbed coral reefs (New Caledonia, South Pacific). J Exp Mar Biol Ecol. 2007;344(1):88–100.

[26] Jolivet A, Chauvaud L, Thébault J, Robson AA, Dumas P, Amos G, et al Circadian behaviour of *Tectus (Trochus) niloticus* in the southwest Pacific inferred from accelerometry. Movement Ecology. 2015;3(1):1–12. 26380713 10.1186/s40462-015-0054-5 PMC4572623

[27] Andréfouët S, Muller-Karger FE, Robinson JA, Kranenburg CJ, Torres-Pulliza D, Spraggins SA, et al., editors. Global assessment of modern coral reef extent and diversity for regional science and management applications: a view from space. 10th Int Coral Reef Symposium; 2006; Okinawa, Japan: Japanese Coral Reef Society.

[28] Kohler KE, Gill SM. Coral Point Count With Excel Extensions (Cpce): a Visual Basic Program for the Determination of Coral and Substrate Coverage Using Random Point Count Methodology. Computers & Geosciences. 2006;32(9):1259–69.

[29] Dumas P, Bertaud A, Peignon C, Leopold M, Pelletier D. A "quick and clean" photographic method for the description of coral reef habitats. J Exp Mar Biol Ecol. 2009;368(2):161–8.

[30] Cinner JE, Aswani S. Integrating customary management into marine conservation. Biol Conserv. 2007;140(3–4):201–16.

[31] Jimenez H, Dumas P, Leopold M, Ferraris J. Invertebrate harvesting on tropical urban areas: Trends and impact on natural populations (New Caledonia, South Pacific). Fish Res. 2011;108(1):195–204.

[32] Ceccarelli DM, Beger M, Kospartov MC, Richards ZT, Birrell CL. Population trends of remote invertebrate resources in a marine reserve: trochus and holothurians at Ashmore Reef. Pac Conserv Biol. 2011;17:132–140.

[33] Merchandise B. Trochus stock assessment project in Emae. Port-Vila: ORSTOM, 1991.

[34] Colquhoun JR. Habitat preferences of juvenile trochus in Western Australia: implications for stock enhancement and assessment. SPC Trochus Information Bulletin. 2001;7:14–20.

[35] Bartlett CY, Manua C, Cinner J, Sutton S, Jimmy R, South R, et al Comparison of Outcomes of Permanently Closed and Periodically Harvested Coral Reef Reserves. Conserv Biol. 2009;23(6):1475–84. 10.1111/j.1523-1739.2009.01293.x 19624531

[36] Underwood AJ. Beyond Baci—the Detection of Environmental Impacts on Populations in the Real, but Variable, World. J Exp Mar Biol Ecol. 1992;161(2):145–78.

[37] Underwood AJ. Beyond Baci—Experimental Designs for Detecting Human Environmental Impacts on Temporal Variations in Natural Populations. Aust J Mar Freshw Res. 1991;42(5):569–87.

[38] Underwood AJ, Chapman MG. Power, precaution, Type II error and sampling design in assessment of environmental impacts. J Exp Mar Biol Ecol. 2003;296(1):49–70.

[39] Dale VH, Beyeler SC. Challenges in the development and use of ecological indicators. Ecological Indicators. 2001;1:3–10.

[40] Benedetti-Cecchi L, Bertocci I, Micheli F, Maggi E, Fosella T, Vaselli S. Implications of spatial heterogeneity for management of marine protected areas (MPAs): examples from assemblages of rocky coasts in the northwest Mediterranean. Mar Environ Res. 2003;55(5):429–58. 10.1016/S0141-1136(02)00310-0 12628195

[41] Leopold M, Cakacaka A, Meo S, Sikolia J, Lecchini D. Evaluation of the effectiveness of three underwater reef fish monitoring methods in Fiji. Biodivers Conserv. 2009;18(13):3367–82.

[42] Sale PF, Cowen RK, Danilowicz BS, Jones GP, Kritzer JP, Lindeman KC, et al Critical science gaps impede use of no-take fishery reserves. Trends Ecol Evol. 2005;20(2):74–80. 10.1016/j.tree.2004.11.007 16701346

[43] Dumas P, Jimenez H, Peignon C, Wantiez L, Adjeroud M. Small-scale habitat structure modulates the effects of no-take marine reserves for coral reef macroinvertebrates. Plos One. 2013;8(3):e58998-e. 23554965 10.1371/journal.pone.0058998 PMC3598913

[44] Hughes TP. Community Structure and Diversity of Coral Reefs—the Role of History. Ecology. 1989;70(1):275–9.

[45] Hughes TP, Connell JH. Multiple Stressors on Coral Reefs: a Long-Term Perspective. Limnol Oceanogr. 1999;44(3):932–40.

[46] Adjeroud M. Factors Influencing Spatial Patterns on Coral Reefs Around Moorea, French Polynesia. Marine Ecology-Progress Series. 1997;159:105–19.

[47] Rao HS. On the habitat and habits of *Trochus niloticus* Linn. in the andaman seas. Rec Indian Mus. 1937;39:47–82.

[48] Long BG, Poiner IR, Harris ANM. Method of Estimating the Standing Stock of *Trochus-Niloticus* Incorporating Landsat Satellite Data, with Application to the Trochus Resources of the Bourke Isles, Torres Strait, Australia. Mar Biol. 1993;115(4):587–93.

[49] Underwood AJ, Chapman MG. Experimental Analyses of the Influences of Topography of the Substratum on Movements and Density of an Intertidal Snail, *Littorina Unifasciata*. J Exp Mar Biol Ecol. 1989;134(3):175–96.

[50] Chapman MG, Underwood AJ. Dispersal of the Intertidal Snail, *Nodilittorina Pyramidalis*, in Response to the Topographic Complexity of the Substratum. J Exp Mar Biol Ecol. 1994;179(2):145–69.

[51] Chapman MG. A comparative study of differences among species and patches of habitat on movements of three species of intertidal gastropods. J Exp Mar Biol Ecol. 2000;244(2):181–201.

[52] Mackay DA, Underwood AJ. Experimental Studies on Homing in Intertidal Patellid Limpet *Cellana Tramoserica* (Sowerby). Oecologia. 1977;30(3):215–37. 10.1007/BF01833629 28309343

[53] Branch GM, Branch ML. Experimental-Analysis of Intraspecific Competition in an Inter-Tidal Gastropod, *Littorina Unifasciata*. Aust J Mar Freshw Res. 1981;32(4):573–89.

[54] Ban NC, Mills M, Tam J, Hicks CC, Klain S, Stoeckl N, et al A social-ecological approach to conservation planning: embedding social considerations. Front Ecol Environ. 2013;11(4):194–202.

[55] Cohen PJ, Steenbergen DJ. Social dimensions of local fisheries co-management in the Coral Triangle. Environ Conserv. 2015;42(3):278–88.

[56] Garcia-Charton JA, Williams ID, Perez-Ruzafa A, Milazzo M, Chemello R, Marcos C, et al Evaluating the ecological effects of Mediterranean marine protected areas: habitat, scale and the natural variability of ecosystems. Environ Conserv. 2000;27(2):159–78.

[57] Garcia-Charton JA, Perez-Ruzafa A, Saanchez-Jerez P, Bayle-Sempere JT, Renones O, Moreno D. Multi-scale spatial heterogeneity, habitat structure, and the effect of marine reserves on Western Mediterranean rocky reef fish assemblages. Mar Biol. 2004;144(1):161–82.

[58] Underwood AJ. The Mechanics of Spatially Replicated Sampling Programs to Detect Environmental Impacts in a Variable World. Aust J Ecol. 1993;18(1):99–116.

[59] Claudet J, Pelletier D. Marine Protected Areas and Artificial Reefs: a Review of the Interactions Between Management and Scientific Studies. Aquat Living Resour. 2004;17(2):129–38.

[60] Kaku R, Ham J, Dumas P, Kaltavara J. Post-Cyclone assessment in Takara village. Port-Vila, Vanuatu: Vanuatu Fisheries Department, 2016.

[61] Govan H. Status and potential of locally-managed marine areas in the South Pacific: meeting nature conservation and sustainable livelihood targets through wide-spread implementation of LMMAs. 2009.

[62] Schiller A, Hunsaker CT, Kane MA, Wolfe AK, Dale VH, Suter GW, et al Communicating ecological indicators to decision makers and the public. Conserv Ecol. 2001;5(1).

[63] Elliott M, Lawrence AJ. The protection of species versus habitats—Dilemmas for marine scientists. Mar Pollut Bull. 1998;36(3):174–6.

[64] Van Wynsberge S, Andrefouet S, Hamel MA, Kulbicki M. Habitats as Surrogates of Taxonomic and Functional Fish Assemblages in Coral Reef Ecosystems: A Critical Analysis of Factors Driving Effectiveness. Plos One. 2012;7(7). 10.1371/journal.pone.0040997 PMC3397997 22815891

[65] Leopold M, Cornuet N, Andrefouet S, Moenteapo Z, Duvauchelle C, Raubani J, et al Comanaging small-scale sea cucumber fisheries in New Caledonia and Vanuatu using stock biomass estimates to set spatial catch quotas. Environ Conserv. 2013;40(4):367–79.

[66] Andrefouet S, Gilbert A, Yan L, Remoissenet G, Payri C, Chancerelle Y. The remarkable population size of the endangered clam Tridacna maxima assessed in Fangatau Atoll (Eastern Tuamotu, French Polynesia) using in situ and remote sensing data. Journal of Marine Science. 2005;62:1037–48.

